# Prognostic and Predictive Potential of CCL5 Expression in Muscle-Invasive Bladder Cancer Patients

**DOI:** 10.3390/ijms25126325

**Published:** 2024-06-07

**Authors:** Cedric Smolka, Markus Eckstein, Rudolf Jung, Verena Lieb, Danijel Sikic, Robert Stöhr, Veronika Bahlinger, Simone Bertz, Astrid Kehlen, Arndt Hartmann, Bernd Wullich, Helge Taubert, Sven Wach

**Affiliations:** 1 Institute of Pathology, University Hospital Erlangen, FAU Erlangen-Nürnberg, 91054 Erlangen, Germany; cedric.smolka@fau.de (C.S.); markus.eckstein@uk-erlangen.de (M.E.); rudolf.jung@uk-erlangen.de (R.J.); robert.stoehr@uk-erlangen.de (R.S.); veronika.bahlinger@uk-erlangen.de (V.B.); simone.bertz@uk-erlangen.de (S.B.); arndt.hartmann@uk-erlangen.de (A.H.); 2 Department of Urology and Pediatric Urology, University Hospital Erlangen, FAU Erlangen-Nürnberg, 91054 Erlangen, Germany; verena.lieb@uk-erlangen.de (V.L.); danijel.sikic@uk-erlangen.de (D.S.); bernd.wullich@uk-erlangen.de (B.W.); sven.wach@uk-erlangen.de (S.W.); 3 Comprehensive Cancer Center EMN, University Hospital Erlangen, FAU Erlangen-Nürnberg, 91054 Erlangen, Germany; 4 Bridge Consortium, 68135 Mannheim, Germany; 5 Molecular Diagnostic Section Unit III, Department of Laboratory Medicine, Halle University Hospital, 06097 Halle (Saale), Germany; astrid.kehlen@uk-halle.de

**Keywords:** CCL5, chemokine, muscle-invasive bladder cancer, prognosis, chemotherapy, tumor cells, immune cells

## Abstract

Bladder cancer (BC) is the 12th most commonly diagnosed cancer worldwide. Although there are several well-established molecular and immunological classifications, prognostic and predictive markers for tumor cells and immune cells are still needed. Using a tissue microarray, we analyzed the expression of the chemokine CC motif ligand 5 (CCL5) by immunohistochemistry (IHC) in 175 muscle-invasive BC samples. The application of a single cutoff for the staining status of tumor cells (TCs; positive vs. negative) and immune cells (ICs; positive vs. negative) revealed 75 patients (42.9%) and 123 patients (70.3%) with CCL5-positive TCs or ICs, respectively. IHC results were associated with prognostic and predictive data. Multivariate Cox regression analysis revealed that positive CCL5 staining in TCs was associated with significantly shorter disease-specific survival (DSS; RR = 1.51; *p* = 0.047), but CCL5-negative ICs were associated with significantly shorter overall survival (OS; RR = 1.66; *p* = 0.005), DSS (RR = 2.02; *p* = 0.001) and recurrence-free survival (RFS; RR = 1.94; *p* = 0.002). Adjuvant chemotherapy was favorable for patients with CCL5-negative ICs for OS (RR = 0.30; *p* = 0.006), DSS (RR = 0.36; *p* = 0.022) and RFS (RR = 0.41; *p* = 0.046) but not for patients with CCL5-positive ICs, except in the subgroup of N1 + N2 patients, where it was associated with better OS. We suggest that CCL5 expression can be a prognostic and predictive marker for muscle-invasive bladder cancer patients.

## 1. Introduction

Bladder cancer (BC) accounts for approximately 3% of global cancer diagnoses. It was recently the 12th most commonly diagnosed cancer and the 14th leading cause of cancer-related death worldwide [1]. Approximately 25% of BC cases are muscle-invasive BC (MIBC) cases [2]. The current therapy for MIBC consists of systemic chemotherapy and/or immunotherapy, radical treatment (cystectomy or radiotherapy), or palliation [3,4]. In addition, trimodality therapy, i.e., maximal endoscopic transurethral resection of the bladder tumor followed by concurrent chemo-radiotherapy as an alternative to radical cystectomy, has been discussed [5]. The degree of lymph node involvement and tumor stage are prognostic factors for MIBC [3,6]. Adjuvant chemotherapy was effective in lymph node-positive MIBC patients regardless of their p53 status [7]. Protein and glycoprotein biomarkers are a demonstrably viable option in BC diagnostics [8]. However, there are still no applied prognostic and/or predictive protein biomarkers for chemotherapy response in MIBC patients.

Many studies, including our own, have reported that the tumor immune microenvironment is also associated with survival [5,9,10,11,12]. In particular, the presence of tumor-infiltrating immune cells, including lymphocytes identified by their protein or gene expression profile, is associated with superior 5-year overall survival (OS) or disease-specific survival (DSS) [9,10,12]. However, immune cells can also express immune checkpoint receptors, such as programmed death 1 (PD-1), which play a role in restraining immune system hyperactivation. Cancer cells can hijack this coinhibitory pathway and escape immune surveillance [13]. Most recently, combination therapy with an antibody–drug conjugate (enfortumab vedotin) directed against a cell surface receptor (nectin-4) and an inhibitor (pembrolizumab) of the immune checkpoint receptor PD-1 resulted in significantly better outcomes than chemotherapy in patients with untreated locally advanced or metastatic urothelial bladder carcinoma [14]. These findings further support the striking role of the immune microenvironment in the prognosis and therapeutic response of MIBC patients.

Chemokines play a major role in the interaction between cancer cells and the immune microenvironment [15,16]. Chemokines have complex functions both in anti-tumor and pro-tumor immune responses, as reviewed in [17]. Recently, we showed that the protein expression of the chemokine CCL2 (monocyte chemotactic protein 1/MCP-1) in tumor cells (TCs) was an independent negative prognostic factor for overall survival (OS), but its expression in immune cells (ICs) was an independent positive prognostic factor for disease-specific survival (DSS) in MIBC patients [18]. A major tumor-promoting role for the coexpression of the chemokines CCL2 and CCL5 in tumor cells has been suggested in breast malignancies [19]. Furthermore, in breast cancer, CCL2 and CCL5 expression is restricted not only to tumor cells but also to cells of the tumor microenvironment, including fibroblasts, endothelial cells, mesenchymal stem cells, smooth muscle cells and immune cells such as tumor-associated macrophages and T cells [20].

The CCL5 gene was first described by Schall et al. [21]. It encodes a T-cell-specific molecule that Schall et al. termed RANTES (an acronym for regulated upon activation, normally T-expressed, and presumably secreted). It belongs to the CC (cysteine–cysteine) motif subfamily of chemokines and is involved in intercellular communication [22,23]. CCL5 is overexpressed in many tumor types, such as breast cancer, pancreatic cancer, colorectal carcinoma, esophageal cancer, prostate cancer, lung cancer, gastric adenocarcinoma, melanoma, head and neck cancer, acute lymphocytic leukemia, Hodgkin lymphoma, multiple myeloma, chondrosarcoma, and osteosarcoma, as reviewed in [24]. Downstream pathways of CCL5 and its main receptor CCR5 include the PI3K/AKT, NF-kB, HIF-a, RAS-ERK-MEK, JAK-STAT and TGF-β-Smad pathways, which are associated with cell proliferation, angiogenesis, apoptosis, invasion, metastasis, and inflammation, as reviewed in [25].

In this study, we investigated whether the chemokine CCL5 is associated with prognosis when expressed in TCs or ICs in MIBC patients and whether this association is comparable to that of the chemokine CCL2. In addition, we were interested in whether CCL5 can be used as a predictive marker for chemotherapy response.

## 2. Results

### 2.1. Correlation of CCL5 Expression with Clinicopathological Parameters and Prognostic Parameters

**Figure 1:**
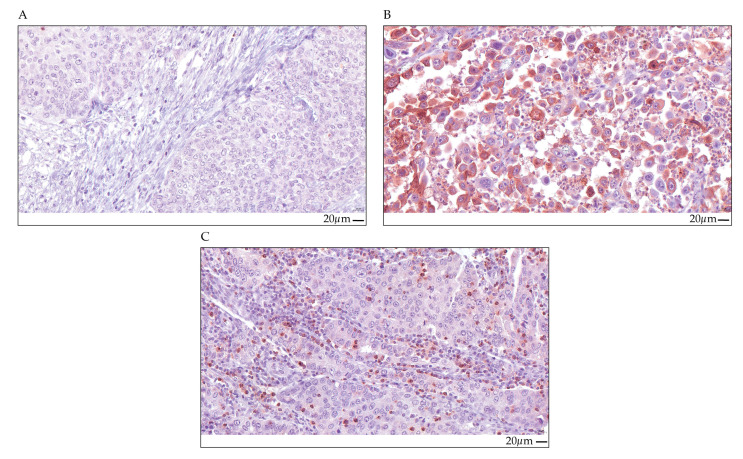
CCL5 immunohistochemical staining in TCs and ICs. Upper row, (**A**) TCs and ICs, CCL5 negative, (**B**) TCs CCL5 positive with IRS = 8 (ICs, negative); lower row, (**C**) ICs CCL5 positive with 20% positivity (TCs, negative). All the images are at 40× magnification, and the scale bar represents 20 µm.

The CCL5 protein expression in a cohort of 175 MIBC patients was studied by immunohistochemistry (IHC) (Figure 1 and Table 1). The clinicopathological data of the patients are summarized in Table 1. CCL5 protein expression was analyzed in tumor cells (TCs) and in immune cells (ICs). CCL5 expression was scored as positive vs. negative for TCs and for ICs in the tumor cell area. We detected 100 patients (57.1%) with no CCL5-stained TCs and 52 patients (29.7%) with no CCL5-stained ICs. In addition, there were 75 patients (42.9%) with CCL5-positive TCs and 123 patients (70.3%) with CCL5-positive ICs (Supplementary Table S1). Representative examples of CCL5 protein expression detected by IHC are shown in Figure 1.

Next, we studied whether CCL5 staining was associated with clinicopathological and molecular parameters by correlation tests (Spearman’s bivariate correlation test).

In TC, CCL5 staining was not associated with sex, tumor stage, chemotherapy treatment, molecular subtype, overall survival (OS), disease-specific survival (DSS), or recurrence-free survival (RFS). A significant positive association was detected with lymph node stage (r_s_ = 0.159; *p* = 0.036) and with CCL5 staining in the IC (r_s_ = 0.184; *p* = 0.015). A significant negative correlation was observed for CCL5 staining with survival time (r_s_ = −0.195; *p* = 0.010) and time to recurrence (r_s_ = −0.163; *p* = 0.031; Supplementary Table S2).

In patients with IC, there was no association between CCL5 staining and age, sex, tumor stage, lymph node stage, chemotherapy treatment, molecular subtype, or OS. CCL5-positive ICs and CCL5-positive TCs (r_s_ = 0.184; *p* = 0.015) were significantly positively associated with survival time (r_s_ = 0.234; *p* = 0.002), time to recurrence (r_s_ = 0.257; *p* = 0.001), DSS (r_s_ = 0.152; *p* = 0.045), and RFS (r_s_ = 0.152; *p =* 0.045). In addition, there was a significant correlation with immune cell subtypes/IC markers, i.e., tumor infiltrating lymphocytes (TILs; r_s_ = 0.368; *p* <0.001), CD3^+^ T cells (r_s_ = 0.301; *p* <0.001), cytotoxic CD8^+^ T cells (r_s_ = 0.348; *p* <0.001), CD56^+^ natural killer cells (NK cells; r_s_ = 0.351; *p* <0.001) and CXCL9 protein expression (r_s_ = 0.394; *p* <0.001). There was no negative correlation with clinicopathological or molecular parameters (Supplementary Table S2).

### 2.2. Association of CCL5 Protein Expression in TCs with Survival

**Figure 2:**
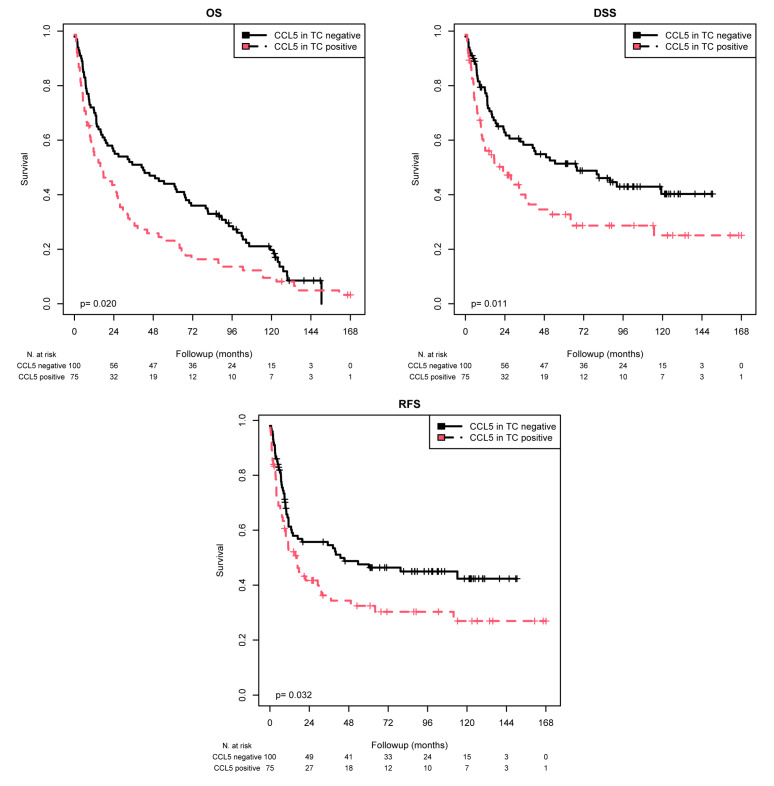
K–M analysis: association between CCL5 expression in TCs and prognosis. Positive CCL5 staining in TCs was associated with a shorter mean OS (*p* = 0.020), mean DSS (*p* = 0.011), and mean RFS (*p* = 0.032) than negative CCL5 staining.

CCL5 staining in the TC was scored as positive or negative by IHC. Kaplan–Meier (K–M) analysis revealed significant associations between positive CCL5 staining and shorter mean OS (*p* = 0.020), mean DSS (*p* = 0.011), and mean RFS (*p* = 0.032) (Table 2 and Figure 2). When comparing the patients with CCL5-positive TC with those with CCL5-negative TC, the mean OS was 36.9 months vs. 56.7 months, the mean DSS was 58.4 vs. 78.7 months, and the mean RFS was 57.7 vs. 75.1 months. According to the univariate Cox regression analysis, CCL5 positivity was associated with a 1.46-fold increased risk of death (*p* = 0.021), a 1.65-fold increased risk of disease-specific death (*p* = 0.012), and a 1.53-fold increased risk of recurrence (*p* = 0.033; Table 3). According to multivariate Cox regression analysis (adjusted for tumor stage, lymph node stage, chemotherapy, and molecular subtype), CCL5 positivity appeared to be an independent poor prognostic factor only for DSS (RR = 1.51; *p* = 0.047; Table 3).

Next, we analyzed the association of CCL5 expression in TCs with prognosis (OS, DSS, RFS) in different patient subgroups (Table 2 and Table 3). K–M analysis revealed that positive CCL5 expression in TCs was associated with shorter OS in the following subgroups: patients who did not receive chemotherapy (*p* = 0.036) and those with tumors of the basal (*p* = 0.028) or luminal (*p* = 0.004) molecular subtypes. Furthermore, positive CCL5 expression in the TC subgroup was associated with shorter DSS in the following subgroups: pT3 + 4 (*p* = 0.046), N0 (*p* = 0.042), no chemotherapy (*p* = 0.035) and luminal tumors (*p* = 0.001). In addition, according to the K–M analysis, positive CCL5 expression in TCs was associated with shorter RFS in patients with luminal-type tumors (*p* = 0.007). Likewise, univariate Cox regression analysis revealed an increased risk of death in the following subgroups: patients who did not receive chemotherapy (RR = 1.47; *p* = 0.037) and patients with tumors of the basal (RR = 1.69; *p* = 0.029) or luminal (RR = 2.09; *p* = 0.005) molecular subtypes. Furthermore, CCL5 positivity in TCs was associated with a 1.55-fold, 1.81-fold, 1.65-fold, and 2.60-fold increased risk of tumor-associated death for patients with tumor stage pT3 + 4, nodal stage N0, patients without chemotherapy or tumors of the luminal molecular subtype, respectively. According to multivariate Cox regression analysis (adjusted for tumor stage, lymph node stage, chemotherapy, and molecular subtype), CCL5 positivity remained an independent prognostic marker for OS in the luminal molecular subtype subgroup (RR = 2.18; *p* = 0.010) and an independent prognostic marker for DSS in the following subgroups: tumor stage pT2 (RR = 3.69; *p* = 0.044), nodal stage N0 (RR = 1.92; *p* = 0.033), and tumors of the luminal molecular subtype subgroup (RR = 2.99; *p* = 0.002). In addition, CCL5 positivity in TCs was an independent prognostic marker for RFS in patients with luminal-type tumors (RR = 2.09; *p* = 0.034; Table 3).

### 2.3. Association of CCL5 Protein Expression in ICs with Survival

For statistical survival analysis, an optimized cutoff for the percentage of CCL5-positive ICs was determined by expert consensus (AH and ME). A background positivity until staining of 6% of ICs (≤6% CCL5-positive ICs) was considered as negative and a staining of more than 6% of ICs (>6% CCL5-positive ICs) was regarded as positive. This grouping is in line with our previous classification of CCL2 staining in ICs [18].

**Figure 3:**
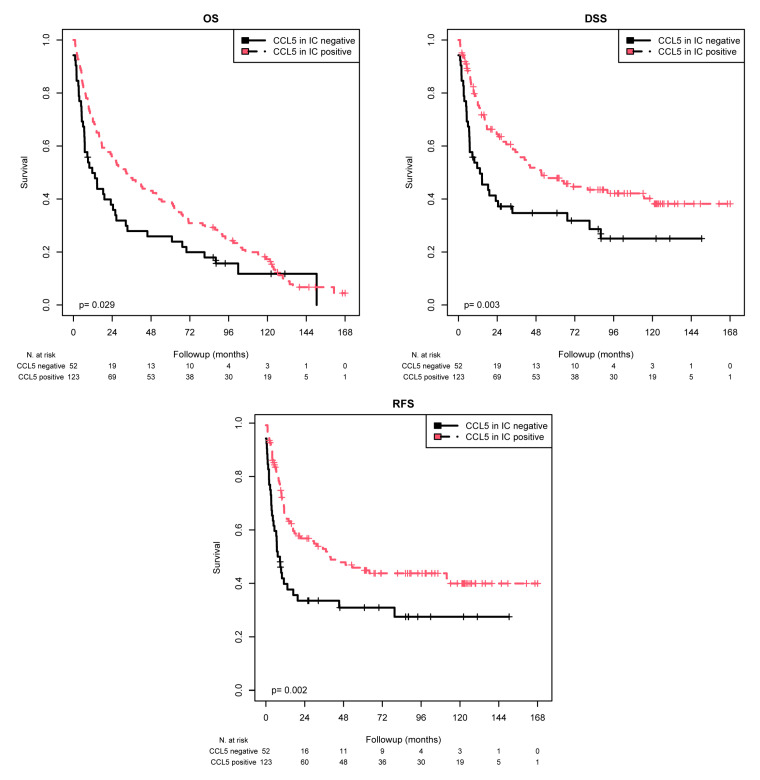
K–M analysis: association between CCL5 expression in ICs and prognosis. Positive CCL5 staining in ICs was associated with a longer mean OS (*p* = 0.029), mean DSS (*p =* 0.003), and mean RFS (*p =* 0.002) than negative CCL5 staining.

K–M analysis revealed significant associations with the mean OS (*p* = 0.029), mean DSS (*p* = 0.003), and mean RFS (*p* = 0.002) (Table 4 and Figure 3). When comparing the patients with CCL5-positive ICs with those with CCL5-negative ICs, the mean OS was 53.9 months vs. 37.4 months, the mean DSS was 82.9 months vs. 51.0 months, and the mean RFS was 81.0 vs. 48.8 months. Univariate Cox regression analysis showed that CCL5 negativity was associated with a 1.47-fold increased risk of death (*p* = 0.030; Table 5), a 1.83-fold increased risk of disease-specific death (*p* = 0.004; Table 5), and a 1.91-fold increased risk of recurrence (*p* = 0.002; Table 5). Cox multivariate regression analysis (adjusted for tumor stage, lymph node stage, chemotherapy, and molecular subtype) revealed that CCL5 staining was an independent predictor of OS (RR = 1.66; *p* = 0.005), DSS (RR = 2.02; *p* = 0.001) and RFS (RR = 1.94; *p* = 0.002; Table 5). Overall, in contrast to TC, CCL5 positivity in ICs appears to be a good prognostic factor for MIBC patients.

Next, we analyzed the association of CCL5 expression in ICs with prognosis (OS, DSS, RFS) in different patient subgroups (Table 4 and Table 5). K–M analysis revealed that positive CCL5 expression in the IC was associated with shorter OS in Nodal stage NX (*p* = 0.035) patients who did not receive chemotherapy (*p* = 0.006). Furthermore, positive CCL5 expression in the IC was associated with shorter DSS in the following subgroups: tumor stage pT3 + 4 (*p* = 0.003), N0 (*p* = 0.037), NX (*p* = 0.035), and no chemotherapy (*p* < 0.001). In addition, according to the K–M analysis, positive CCL5 expression in the IC was associated with shorter RFS in the following subgroups: tumor stage 3 + 4 (*p* < 0.001), N0 (*p* = 0.030), N1/N2 (*p* = 0.039) and no chemotherapy (*p* < 0.001). Accordingly, univariate Cox regression analysis revealed an increased risk of death in the Nodal stage NX subgroup (RR = 3.52, *p* = 0.045) and in patients who did not receive chemotherapy (RR = 1.74; *p* = 0.006). Furthermore, CCL5 positivity in ICs was associated with a 1.93-fold, 1.87-fold, 3.52-fold, and 2.21-fold increased risk of tumor-associated death for patients with tumor stage pT3 + 4, nodal stage N0, nodal stage NX, or no chemotherapy, respectively. According to the multivariate Cox regression analysis (adjusted for tumor stage, lymph node stage, chemotherapy, and molecular subtype), CCL5 positivity remained an independent prognostic marker for OS in the following subgroups: tumor stage pT3 + 4 (RR = 1.70; *p* = 0.010) and no chemotherapy (RR = 1.83; *p* = 0.004), and it was an independent prognostic marker for DSS in the following subgroups: tumor stage pT3 + 4 (RR = 2.31; *p* < 0.001) and no chemotherapy (RR = 2.36; *p* < 0.001). In addition, CCL5 positivity in the IC was an independent prognostic marker for RFS in the following subgroups: tumor stage pT3 + 4 (RR = 2.38; *p* < 0.001) and no chemotherapy (RR = 2.32; *p* = 0.001). Overall, CCL5 positivity in the IC was an independent prognostic marker for OS, DSS and RFS in all MIBC patients and in the two subgroups, namely, patients with pT3 + 4 tumors and patients who did not receive chemotherapy.

### 2.4. Association of Chemotherapy Response and CCL5 Protein Expression in ICs

To test for an association between CCL5 expression in ICs and chemotherapy response, patients with positive or negative CCL5 expression in ICs were separately considered in the following subgroups: tumor stage, nodal stage, and molecular subtype.

**Figure 4:**
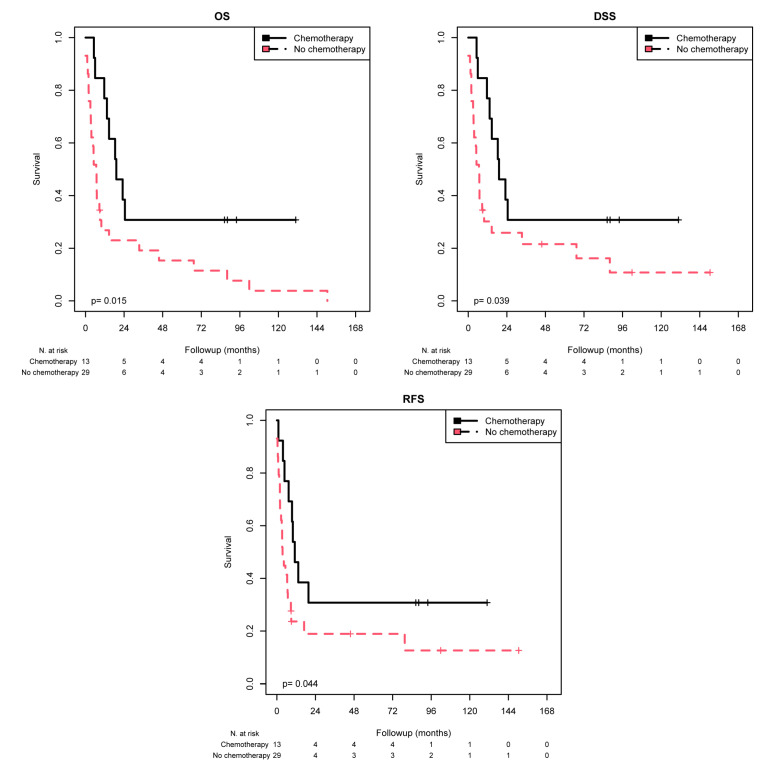
K–M analysis: association of chemotherapy with prognosis (OS, DSS, and RFS) in the tumor stage 3 + 4 group stratified by negative CCL5 staining in the IC. In the tumor stage 3 + 4 group with negative CCL5 IC staining, chemotherapy treatment was associated with longer OS (*p* = 0.004), longer DSS (*p* = 0.011) and longer RFS (*p* = 0.024).

K–M analysis revealed a significant association between chemotherapy response and CCL5-negative ICs for the tumor stage subgroup pT3 + 4 in terms of the mean OS (*p* = 0.015), mean DSS (*p* = 0.039), and mean RFS (*p* = 0.044) (Table 6 and Figure 4). In other words, patients with pT3 + 4 and CCL5-negative ICs had a better prognosis after chemotherapy treatment than patients without such treatment, as reflected by a prolonged OS (50.7 vs. 22.4 months), DSS (50.7 vs. 29.6 months) and RFS (46.4 vs. 27.3 months). In addition, patients in the subgroup of N1/N2 had better OS after chemotherapy (mean 46.6 months) than did those who did not receive chemotherapy (mean 18.9 months; *p* = 0.024).

Univariate Cox regression analysis showed that chemotherapy treatment in the subgroup with a tumor stage of pT3 + 4 with CCL5-negative ICs resulted in a 0.40-fold reduced risk of death (*p* = 0.019), a 0.45-fold reduced risk of disease-specific death (*p* = 0.044), and a 0.46-fold reduced risk of recurrence (*p* = 0.049; Table 7). In addition, in the subgroup of N1/N2 patients, those with CCL5-positive ICs had a 0.45-fold lower risk of death *(p* = 0.028) after chemotherapy than patients who did not receive chemotherapy.

Multivariate Cox regression analysis (adjusted for tumor stage, lymph node stage, chemotherapy, and molecular subtype) revealed that chemotherapy treatment in all patients with CCL5-negative ICs remained an independent predictor of OS (RR = 0.30; *p* = 0.006), DSS (RR = 0.36; *p* = 0.022), and RFS (RR = 0.41; *p* = 0.046; Table 7) compared to patients who did not receive chemotherapy. Similarly, this prognostic effect was also found for OS (RR = 0.28; *p* = 0.004), DSS (RR = 0.32; *p* = 0.011), and RFS (RR = 0.36; *p* = 0.024) in the CCL5 IC-negative subgroup of patients in the tumor stage pT3 + 4 subgroup for OS (RR = 0.25; *p* = 0.023), DSS (RR = 0.29; *p* = 0.041), and RFS (RR = 0.23; *p* = 0.020). In addition, in CCL5-negative IC patients in the nodal stage N0 subgroup, chemotherapy treatment was associated with a 0.22-fold reduced risk of death (*p* = 0.028). However, in the CCL5 IC-positive patients in the nodal stage N1/N2 subgroup, chemotherapy treatment was associated with a 0.44-fold reduced risk of death (*p* = 0.026) compared to the patients in this subgroup who did not receive chemotherapy.

Overall, chemotherapy treatment is associated with a better prognosis mostly in CCL5-negative patients but also in patients in the N1/N2 subgroup, where CCL5 IC-positive patients benefit from chemotherapy treatment.

## 3. Discussion

In our study, we analyzed the protein expression of CCL5 in TCs and ICs in MIBC patients (n = 175) and assessed its association with clinicopathological and survival data for the first time. Interestingly, CCL5 staining in TCs was weakly positively correlated with CCL5 staining in ICs. Remarkably, CCL5 staining in TCs was positively correlated with the lymph node stage and negatively correlated with survival time and time to recurrence. CCL2 expression in TCs was also correlated with time to recurrence [18]. In contrast, CCL5 staining in ICs was positively correlated with survival time, time to recurrence, DSS, and RFS. As expected, we observed a strong correlation between immune cell markers and CCL5 expression in ICs. The highest correlations were detected with markers for NK cells and cytotoxic CD8+ cells, which is in agreement with the positive correlation with survival time and time to recurrence in this study and our previous study, where the cytotoxic T-cell-related gene expression signature predicted improved survival in MIBC patients [9]. In addition, we reported comparable correlations of CCL2 expression in ICs with DSS and RFS [18]. This concordance suggests a somewhat coordinated coexpression of the chemokines CCL2 and CCL5 in MIBC, as has been reported in breast cancer [20].

Next, we studied whether CCL5 expression in TCs or in ICs was an independent prognostic marker in all MIBC patients or in subgroups of MIBC patients stratified according to tumor stage, lymph node stage, chemotherapy treatment or molecular subtype.

Multivariate Cox regression analysis showed that CCL5 positivity in TCs was an independent negative prognostic marker for DSS in all MIBC patients. In addition, CCL5 positivity in TCs was an independent prognostic factor for OS, DSS and RFS in MIBC patient subgroups, i.e., in the luminal molecular subtype subgroup. In addition, DSS in the tumor stage 2 and nodal stage N0 subgroups was analyzed. In our previous study of CCL2 expression in the TC of MIBC patients, we found that CCL2 positivity in TCs of the luminal molecular subtype was associated with shorter OS [18]. In contrast to the previous study, in all patients and in tumor stage 2 patients, CCL2 positivity in TC was associated with shorter OS, while in the chemotherapy-treated patients, CCL2 positivity was associated with shorter OS and DSS. These findings suggest that, although CCL5 and CCL2 positivity in TCs is a negative prognostic factor, this finding can be applied to different MIBC patient groups and prognostic outcomes.

In contrast to the findings in TCs, CCL5 positivity in ICs was an independent positive prognostic parameter for OS, DSS, and RFS in all MIBC patients. In addition, CCL5 positivity was also an independent prognostic parameter in the MIBC subgroups, i.e., for OS, DSS, and RFS in both the tumor stage 3 + 4 and no chemotherapy subgroups. In our previous study of CCL2 expression in ICs, we found that CCL2 IC positivity was associated with a longer DSS for all MIBC patients and for those from the subgroup without chemotherapy. The latter subgroup also exhibited CCL2 positivity in ICs and a longer RFS. However, again somewhat different in the present study, CCL5 positivity in ICs was associated with longer OS, DSS, and RFS, which was not found in a previous study [18]. This finding suggested that although CCL5 and CCL2 positivity in ICs is a positive prognostic factor, this finding can be applied to different MIBC patient groups and prognostic outcomes.

There are different prognostic impacts of CCL5 staining in TCs and ICs. The CCL5 axis and its main receptor CCR5 support tumor progression through multiple mechanisms, such as increasing tumor growth, inducing extracellular matrix remodeling, enhancing tumor cell migration (metastasis formation), expanding cancer cell stemness, promoting cancer cell resistance to drugs, decreasing cytotoxicity to DNA-damaging agents, deregulating cellular energetics (metabolic reprogramming), and promoting angiogenesis, as reviewed in [24]. Breast cancer cells can stimulate the de novo secretion of CCL5 from mesenchymal stem cells within the tumor stroma, which then acts in a paracrine fashion on cancer cells to enhance their motility, invasion, and metastasis [26]. The oncogene MYC, which functions as a transcription factor in many human tumors, elicits the production of chemokines, including CCL2 and CCL5. This attracts inflammatory cells (e.g., mast cells), which promote angiogenesis and tumor growth [27]. NF-κB activity in breast cancer mouse cells can induce the expression of CCL5, which drives the recruitment of CCR5-expressing macrophages, which supplies breast tumor cells with collagen that promotes their proliferation [28]. Therapy-induced changes in the expression of chemokines can contribute to tumor resistance or tumor recurrence. The upregulation of CCL2 and CCL5 postradiotherapy results in the recruitment of immunosuppressive cells, such as CCR2 + CCR5+ monocytes, MDSCs, and CCR2+ Treg cells, leading to cancer outgrowth, as reviewed in [17].

On the other hand, CCL5 is a natural adjuvant for enhancing anti-tumor immune responses [29]. CCL5 promotes anti-tumor immunity by recruiting anti-tumor T cells and dendritic cells to the tumor microenvironment, and, in this way, it increases the immunotherapy response in different tumor types, as reviewed in [24]. Together with IL-2 and IFN-γ, which are released by T cells, CCL5 induces the activation and proliferation of particular NK cells to generate C-C chemokine-activated killer cells [20,30]. Conversely, stimulated NK cells can produce T-cell-recruiting chemokines, including CCL2 and CCL5, in breast cancer patients [31].

Our interesting finding of a response to chemotherapy in CCL5-negative IC patients led us to study the chemotherapy response—analyzed by OS, DSS, and RFS—separately in patients with CCL5-positive or CCL5-negative ICs in all subgroups of MIBC patients and the complete MIBC patient cohort. Chemotherapy treatment in all patients, but only in those with CCL5-negative ICs, was an independent positive predictor of OS, DSS and RFS compared to patients who did not receive chemotherapy. In the MIBC subgroup analysis this prognostic effect was also found for OS, DSS, and RFS in CCL5 IC-negative patients in the tumor stage 3 + 4 subgroup or in the luminal molecular subtype subgroup. Furthermore, in IC CCL5-negative patients in the nodal stage N0 subgroup, chemotherapy treatment was associated with a reduced risk of death. However, in CCL5-positive patients in the nodal stage N1/N2 subgroup, chemotherapy treatment was associated with a reduced risk of death compared to patients who did not receive chemotherapy. We suggest that CCL5 could mark a population of ICs that may be anti-tumorigenic in N0 patients but pro-tumorigenic in N1 + 2 MIBC patients. Generally, a tumor, such as bladder cancer, is a key immunological player that can shape immune responses to favor itself [32]. For an overview of tumor-infiltrating immune cells and their therapeutic implications, we would like to refer to excellent reviews [17,33]. A Swedish study of MIBC reported that patients treated with radio-/chemotherapy or radiotherapy had better OS and DSS than untreated patients [34]; however, in that study, no further stratification (e.g., for immunological markers) was performed.

Overall, chemotherapy was associated with a better prognosis, mostly in CCL5-negative patients. However, chemotherapy was not advantageous for OS in all CCL5 IC-positive patients but was beneficial for OS in the N1/N2 subgroup. In our previous study on the effect of chemotherapy on CCL2 staining in ICs, we found that, in the subgroup with the most aggressive tumors (N1 + 2 and tumor stage 3+ 4), patients with CCL2-positive ICs showed a better response to chemotherapy treatment in terms of OS, DSS, and RFS than MIBC patients indicated with negative IC CCL2 staining [35]. However, in our previous CCL2 study, chemotherapy was associated with a poorer prognosis (shorter RFS) in patients with CCL2-positive ICs in the N0 subgroup and with poorer survival (shorter OS, DSS, RFS) in patients with CCL2-positive ICs in the pT2 subgroup than in patients who did not receive chemotherapy. Such a significant association between CCL5 IC positivity and poor survival after chemotherapy could be observed only as a trend for nodal stage N0 patients.

We previously showed that a cytotoxic T-cell-related gene expression signature predicts improved survival in MIBC patients after radical cystectomy and adjuvant chemotherapy [9]. In addition, patients with T-cell-inflamed tumors that are enriched in T-cell-recruiting chemokines, such as CCL5, CXCL9, CXCL10, and CXCL11, are most likely to benefit from checkpoint blockade therapy [17,36].

Our finding that MIBC patients with CCL5-positive ICs have a better prognosis (OS, DSS and RFS) suggests that CCL5 expression in ICs, possibly as a surrogate marker for cytotoxic CD8+ T cells and NK cells, may play a role in the anti-tumor immune response. CD8+ T cells can secrete inflammatory chemokines, such as CCL3 and CCL5, which increase infiltration of neutrophils, monocytes, and Th1 lymphocytes. In this way, they can contribute to a so-called auto-recruitment of cytotoxic T cells [37]. Recently, Sun et al. indicated that CD8+ T cells infiltration can be regulated by a circular RNA (circMGA) that stabilizes CCL5 mRNA in bladder cancer [38]. Interestingly, treatment with circMGA and anti-PD-1 can synergistically suppress xenograft bladder cancer growth [38].

There are different hurdles with CCL5/CCR5 inhibition in tumor therapy since CCL5 acts as a double-edged sword—initially fueling tumor development but also recruiting antitumor cell populations to the tumor over time [39]. For a recent overview of the CCL5/CCR5 network and their clinical application as drug targets, especially in colorectal cancer, we would like to refer to a review by Zhang et al. [40]. In addition, recent findings by Jacobs et al. are of interest. The suppression of CCL5 expression by heat shock Factor 1 (HSF1) prevents CD8+ T-cell influx, which supports immune-mediated tumor killing [41]. The authors suggest that targeting HSF1 could improve immunotherapies. Furthermore, the T-cell–inflamed gene expression profile, which includes CCL5, appears to be an emerging predictive biomarker for the pembrolizumab response [36].

Our study has several limitations. It was a retrospective study, and for a comprehensive statistical analysis of two parameters in eight subgroups, the number of study patients was rather low. In addition, only 24.6% (43/175) of our patients were treated with chemotherapy, which is again a rather small cohort. Ultimately, our results must be evaluated in a larger prospective study. However, altogether, the number of study patients (n = 175) allowed for reasonable multivariate analysis of one parameter, such as CCL5 staining, for its prognostic and predictive relevance in MIBC patients.

Overall, CCL5 positivity in TCs is an independent negative prognostic factor for DSS. In contrast, CCL5 positivity in ICs was significantly associated with improved OS, DSS, and RFS. Chemotherapy treatment was associated with a better prognosis for OS, mostly in CCL5 IC-negative patients, but not in CCL5 IC-positive patients in the N1/N2 subgroup (n = 38). We suggest that CCL5 staining in TCs and ICs seems to be a prognostic marker; additionally, CCL5 detection in ICs might serve as a predictive marker for adjuvant chemotherapy and, possibly, for future immune checkpoint therapy.

## 4. Materials and Methods

### 4.1. Patients and Tumor Materials

Tissue microarrays (TMAs) with formalin-fixed and paraffin-embedded tumor samples from 175 MIBC patients were investigated in this study. The TMA was prepared and comprises the majority of patients described previously [18]. The research carried out on human subjects complied with the Helsinki Declaration. All patients provided written informed consent. The study was approved by the Ethics Commission of the University Hospital Erlangen (No. 3755 and No. 329_16B). Tumor histology was reviewed by two uropathologists (AH, ME). An overview of the clinicopathological parameters of the patients included in this study is given in Table 1.

### 4.2. Immunohistochemistry

Staining for tumor-infiltrating lymphocytes (TILs), CD3+ T cells, cytotoxic CD8+ T cells, CD56, and CXCL9 was performed as previously described [9,18]. For the study of CCL5 protein expression, a manual IHC protocol was applied as previously described [42]. Briefly, after heat pretreatment at 120 °C for 5 min with TE buffer (pH 9) and peroxidase blocking (Dako, Hamburg, Germany), primary antibodies against CCL5 (rabbit polyclonal RANTES antibody, Cat. No. ab9679, dilution 1:200; Abcam, Cambridge, UK) were applied for 30 min.

The stained specimens were viewed at objective magnifications of ×100 and ×200. Negative control slides without the addition of a primary antibody were included for each staining experiment. From each sample, two cores from the center and two cores from the invasive front were analyzed. Afterward, the average staining intensity of both cores was determined since we did not observe significant differences between the two locations.

The expression of CCL5 was detected in TCs, and both cytoplasmic and nuclear staining were considered (average of stained TCs in the invasion front and in the tumor center) and characterized as positive or negative. In terms of ICs (average of stained ICs in the invasion front and the tumor center), they were considered in terms of the percentage of CCL5-positive ICs out of all ICs. There were no relevant differences in staining intensities, so only those that were positive or negative for TC and the percentage of CCL5-positive ICs exclusively were counted. For the survival analysis, patients were grouped as CCL5-positive vs. CCL5-negative in TCs and ≤6% CCL5-positive ICs vs. >6% CCL5-positive ICs. Slides were scanned with a P250 slide scanner (3DHistech, Budapest, Hungary) and analyzed using CaseViewer2.0 (3DHistech). Photos were taken with a Leica DM 4000B microscope with a 20× HC PL Fluotar objective (Leica, Wetzlar, Germany) and with a Jenoptik Gryphax Arktur camera (Jenoptik AG, Jena, Germany).

### 4.3. Molecular Subtyping via NanoString Technology

RNA was isolated and purified, as described previously [10]. We selected 21 genes that are known to be stable markers of luminal and basal differentiation according to the MDDACC subtyping approach [10,43,44]. Gene counts were normalized using two reference genes (SDHA and HPRT1) and log2-transformed for further analysis with nSolver 4.0 software.

### 4.4. Statistical Analyses

The associations between the IHC and clinicopathological data were calculated using Spearman’s correlation test, the chi-squared test, or the Mann–Whitney test. The associations of CCL5 expression with OS, DSS, and RFS were determined via univariate analyses (Kaplan–Meier analysis and Cox regression hazard models) and multivariate Cox regression analyses. Multivariate Cox regression analyses were adjusted for parameters that were significantly associated with prognosis, i.e., tumor stage, lymph node stage, chemotherapy, and molecular subtype, as previously described [18]. A *p*-value less than 0.05 was considered to indicate statistical significance. Statistical analyses were performed with the SPSS 22.0.0.0 software package (SPSS Inc., Chicago, IL, USA) and with R V3.2.1 (The R Foundation for Statistical Computing, Vienna, Austria).

## 5. Conclusions

A positivity for CCL5 in TCs was an independent negative prognostic factor for DSS in MIBC patients. In contrast, positivity for CCL5 in ICs appeared as an independent positive prognostic factor for OS, DSS, and RFS in MIBC patients. Importantly, treatment with adjuvant chemotherapy was favorable for MIBC patients with CCL5-negative ICs for OS, DSS, and RFS but not for patients with CCL5-positive ICs, with the exception of the subgroup of N1 + N2 patients, where adjuvant chemotherapy was associated with better OS. CCL5 together with its main receptor CCR5 might provide promising targets, possibly in combination with immunotherapies for MIBC patients. Summarizing, we suggest that CCL5 expression could be a prognostic and predictive marker for MIBC patients.

## Figures and Tables

**Table 1:** Clinicopathological data and survival parameters of the MIBC patients.

Clinicopathological and Survival Parameters	Patients (Percentage)
**Total**	175
**Morphology**	
NOS	90 (51.4)
Squamous	41 (23.4)
Sarcomatoid	9 (5.2)
MPUC	9 (5.2)
PUC	6 (3.4)
Pure neuroendocrine	8 (4.6)
Other rare subtypes	12 (6.8)
**Sex**	
Female	48 (27.4)
Male	127 (72.6)
**Age (years)**	
Range	37.0–91.0
Mean	69.6
Median	71.0
**Tumor Stage**	
pT2	46 (26.3)
pT3	85 (48.6)
pT4	44 (25.1)
**Tumor Grade 1973**	
G2	6 (3.4)
G3	169 (96.6)
**Tumor Grade 2016**	
High grade	175
**Nodal Stage**	
pN0	110 (62.9)
pN1 + 2	52 (29.7)
pNX	13 (7.4)
**Adjuvant Chemotherapy (Ct)**	
Yes	43 (24.6)
No	132 (75.4)
**Survival/observation Time (months)**	
Range	0–168.1
Mean	46.0
Median	24.5
**Overall Survival (OS)**	
Alive	18 (10.3)
Dead	157 (89.7)
**Disease-Specific Survival (DSS)**	
Alive	74 (42.3)
Dead	101 (57.7)
**Recurrence-Free Survival Time (months)**	
Range	0–168.1
Mean	42.2
Median	15.4
**Recurrence-Free Survival (RFS)**	
Without recurrence	74 (42.3)
With recurrence	101 (57.7)
**Molecular Subtypes**	
Basal	80 (45.7)
Double negative	9 (5.1)
Luminal	68 (38.9)
Luminal EMT-p53-like	17 (9.7)
unknown	1 (0.6)

NOS: not otherwise specified; MPUC: micropapillary urothelial carcinoma; PUC: plasmacytoid urothelial carcinoma.

**Table 2:** K–M analysis: association of CCL5 staining in TCs with mean OS, mean DSS, or mean RFS.

Parameter			Kaplan–Meier Analysis				
CCL5	N	OS		DSS		RFS	
Positive vs. Negative							
		Months	*p*	Months	*p*	Months	*p*
**All Patients**	175	36.9 vs. 56.7	**0.020**	58.4 vs. 78.7	**0.011**	57.7 vs. 75.1	**0.032**
**Tumor Stage 2**	46		n.s.	85.4 vs. 115.6	0.050		n.s.
**Tumor Stage 3 + 4**	129	29.5 vs. 47.7	0.050	48.1 vs. 64.8	**0.046**	48.2 vs. 61.2	0.080
**Nodal Stage N0**	110		n.s.	83.2 vs. 101.7	**0.042**	82.4 vs. 99.1	0.072
**Nodal Stage N1/N2**	52		n.s.		n.s.		n.s.
**Nodal Stage NX**	13		n.s.		n.s.		n.s.
**CT−**	132	37.4 vs. 55.9	0.036	64.2 vs. 82.7	**0.035**	64.0 vs. 80.4	0.069
**CT+**	43		n.s.		n.s.		n.s.
**Basal**	80	37.4 vs.68.6	**0.028**	70.5 vs. 94.0	0.093		n.s.
**Luminal**	68	27.7 vs. 56.0	**0.004**	31.3 vs. 74.9	**0.001**	26.7 vs. 69.5	**0.007**
**Luminal EMT-p53-like**	17		n.s.		n.s.		n.s.

Significant *p*-values are marked in bold face; n.s.: not significant.

**Table 3:** Univariate and multivariate Cox regression analyses: Association of CCL5 staining in TCs with mean OS, mean DSS, or mean RFS.

Parameter			Univariate Cox Regression Analysis				
CCL5	N	OS		DSS		RFS	
Positive vs. Negative							
		RR	*p*	RR	*p*	RR	*p*
**All Patients**	175	1.46	**0.021**	1.65	**0.012**	1.53	**0.033**
**Tumor Stage 2**	46		n.s.	2.55	0.058		n.s.
**Tumor Stage 3 + 4**	129	1.44	0.051	1.55	**0.048**	1.46	0.082
**Nodal Stage N0**	110		n.s.	1.81	**0.045**	1.69	0.075
**Nodal Stage N1/N2**	52		n.s.		n.s.		n.s.
**Nodal Stage NX**	13		n.s.		n.s.		n.s.
**CT−**	132	1.47	**0.037**	1.65	**0.037**	1.54	0.072
**CT+**	43		n.s.		n.s.		n.s.
**Basal**	80	1.69	**0.029**	1.72	0.098		n.s.
**Luminal**	68	2.09	**0.005**	2.60	**0.002**	2.19	**0.009**
**Luminal EMT-p53-like**	17		n.s.		n.s.		n.s.
			**Multivariate Cox Regression Analysis**				
**CCL5**	**N**	**OS**		**DSS**		**RFS**	
positive vs. negative							
		**RR**	***p***	**RR**	***p***	**RR**	***p***
**All Patients**	175		n.s.	1.51	**0.047**		n.s.
**Tumor Stage 2**	46		n.s.	3.69	**0.044**	3.46	0.059
**Tumor Stage 3 + 4**	128		n.s.		n.s.		n.s.
**Nodal Stage N0**	109		n.s.	1.92	**0.033**	1.80	0.054
**Nodal Stage N1/N2**	52		n.s.		n.s.		n.s.
**Nodal Stage NX**	13		n.s.		n.s.		n.s.
**CT−**	131		n.s.	1.58	0.074		n.s.
**CT+**	43	2.34	0.057	2.58	0.051		n.s.
**Basal**	80		n.s.		n.s.		n.s.
**Luminal**	68	2.18	**0.010**	2.99	**0.002**	2.09	**0.034**
**Luminal EMT-p53-like**	17		n.s.		n.s.		n.s.

Significant *p*-values are marked in bold face; n.s.: not significant.

**Table 4:** K–M analysis: association of CCL5 staining in ICs with mean OS, mean DSS, or mean RFS.

Parameter			Kaplan–Meier Analysis				
CCL5	N	OS		DSS		RFS	
Negative vs. Positive							
		Months	*p*	Months	*p*	Months	*p*
**All Patients**	175	37.4 vs. 53.9	**0.029**	51.0 vs. 82.9	**0.003**	48.8 vs. 81.0	**0.002**
**Tumor Stage 2**	46		n.s.		n.s.		n.s.
**Tumor Stage 3 + 4**	129	31.7 vs. 44.3	0.098	37.8 vs. 70.2	**0.003**	34.7 vs. 68.6	**<0.001**
**Nodal Stage N0**	110		n.s.	75.5 vs. 109.8	**0.037**	73.3 vs. 108.1	**0.030**
**Nodal Stage N1/N2**	52	11.8 vs. 29.1	0.093		n.s.	8.3 vs. 36.1	**0.039**
**Nodal Stage NX**	13	5.6 vs. 17.6	**0.035**	5.6 vs. 17.6	**0.035**	3.4 vs. 7.8	0.064
**CT−**	132	31.9 vs. 55.9	**0.006**	48.3 vs. 91.4	**<0.001**	47.1 vs. 89.9	**<0.001**
**CT+**	43		n.s.		n.s.		n.s.
**Basal**	80		n.s.	60.0 vs. 92.7	0.086	59.2 vs. 91.8	0.067
**Luminal**	68		n.s.		n.s.		n.s.
**Luminal EMT-p53-like**	17		n.s.		n.s.		n.s.

Significant *p*-values are marked in bold face; n.s.: not significant.

**Table 5:** Univariate and multivariate Cox regression analyses: association of CCL5 staining in ICs with mean OS, mean DSS, or mean RFS.

Parameter			Univariate Cox Regression Analysis				
CCL5	N	OS		DSS		RFS	
Negative vs. Positive							
		RR	*p*	RR	*p*	RR	*p*
**All Patients**	175	1.47	**0.030**	1.83	**0.004**	1.91	**0.002**
**Tumor Stage 2**	46		n.s.		n.s.		n.s.
**Tumor Stage 3 + 4**	129		n.s.	1.93	**0.003**	2.11	**<0.001**
**Nodal Stage N0**	110		n.s.	1.87	**0.040**	1.92	**0.033**
**Nodal Stage N1/N2**	52	1.73	0.098	1.94	0.059	1.91	0.058
**Nodal Stage NX**	13	3.52	**0.045**	3.52	**0.045**	2.99	0.076
**CT−**	132	1.74	**0.006**	2.21	**0.001**	2.28	**<0.001**
**CT+**	43		n.s.		n.s.		n.s.
**Basal**	80		n.s.	1.86	0.092	1.93	0.073
**Luminal**	68		n.s.		n.s.		n.s.
**Luminal EMT-p53-like**	17		n.s.		n.s.		n.s.
			**Multivariate Cox Regression Analysis**				
**CCL5**	**N**	**OS**		**DSS**		**RFS**	
0 vs. >0							
		**RR**	***p***	**RR**	***p***	**RR**	***p***
**All patients**	174	1.66	**0.005**	2.02	**0.001**	1.94	**0.002**
**Tumor Stage 2**	46		n.s.		n.s.		n.s.
**Tumor Stage 3 + 4**	128	1.70	**0.010**	2.31	**<0.001**	2.38	**<0.001**
**Nodal Stage N0**	109		n.s.		n.s.	1.76	0.084
**Nodal Stage N1/N2**	52		n.s.	1.91	0.075	1.97	0.057
**Nodal Stage NX**	13	4.64	0.072	4.64	0.072	4.64	0.061
**CT−**	131	1.83	**0.004**	2.36	**<0.001**	2.32	**0.001**
**CT+**	43		n.s.		n.s.		n.s.
**Basal**	80		n.s.	1.87	0.097	1.92	0.088
**Luminal**	68	1.64	0.095	1.76	0.085	1.73	0.098
**Luminal EMT-p53-like**	17		n.s.		n.s.		n.s.

Significant *p*-values are marked in bold face; n.s.: not significant.

**Table 6:** K–M analysis: association of chemotherapy stratified by CCL5 staining in ICs with mean OS, mean DSS, or mean RFS.

Parameter CT+ vs. CT−				Kaplan–Meier Analysis				
		N	OS		DSS		RFS	
			Months	*p*	Months	*p*	Months	*p*
**All Patients**	**CCL5−**	52		n.s.		n.s.		n.s.
**All Patients**	**CCL5+**	123		n.s.	60.3 vs. 91.4	0.067	55.1 vs. 89.9	0.052
**Tumor Stage 2**	**CCL5−**	10		n.d.		n.d.		n.d.
**Tumor Stage 2**	**CCL5+**	36		n.s.		n.s.		n.s.
**Tumor Stage 3 + 4**	**CCL5−**	42	50.7 vs. 22.4	**0.015**	50.7 vs. 29.6	**0.039**	46.4 vs. 27.3	**0.044**
**Tumor Stage 3 + 4**	**CCL5+**	87		n.s.		n.s.		n.s.
**Nodal Stage N0**	**CCL5−**	32	80.9 vs. 45.9	0.081		n.s.		n.s.
**Nodal Stage N0**	**CCL5+**	78		n.s.	75.1 vs. 116.6	0.068	74.9 vs. 115.3	0.077
**Nodal Stage N1/N2**	**CCL5−**	14		n.s.		n.s.		n.s.
**Nodal Stage N1/N2**	**CCL5+**	38	46.6 vs. 18.9	**0.024**		n.s.		n.s.
**Nodal Stage NX**	**CCL5−**	6		n.s.		n.s.		n.s.
**Nodal Stage NX**	**CCL5+**	7		n.s.		n.s.		n.s.
**Basal**	**CCL5−**	16		n.s.		n.s.		n.s.
**Basal**	**CCL5+**	64		n.s.		n.s.		n.s.
**Luminal**	**CCL5−**	24		n.s.		n.s.		n.s.
**Luminal**	**CCL5+**	44		n.s.	41.7 vs. 71.2	0.099	32.9 vs. 67.4	0.089
**Luminal EMT-p53-like**	**CCL5−**	6		n.s.		n.s.		n.s.
**Luminal EMT-p53-like**	**CCL5+**	11		n.s.		n.s.		n.s.

Significant *p*-values are marked in bold face; n.s.: not significant; n.d.: not determined.

**Table 7:** Univariate and multivariate Cox regression analyses: association of chemotherapy stratified by CCL5 staining in ICs with mean OS, mean DSS, or mean RFS.

Parameter CT+ vs. CT−			Univariate Cox Regression Analysis					
		N	OS		DSS		RFS	
			RR	*p*	RR	*p*	RR	*p*
**All Patients**	**CCL5−**	52		n.s.		n.s.		n.s.
**All Patients**	**CCL5+**	123		n.s.	1.61	0.069	1.66	0.055
**Tumor Stage 2**	**CCL5−**	10		n.d.		n.d.		n.d.
**Tumor Stage 2**	**CCL5+**	36		n.s.		n.s.		n.s.
**Tumor Stage 3 + 4**	**CCL5−**	42	0.40	**0.019**	0.45	**0.044**	0.46	**0.049**
**Tumor Stage 3 + 4**	**CCL5+**	87		n.s.		n.s.		n.s.
**Nodal Stage N0**	**CCL5−**	32	0.35	0.095		n.s.		n.s.
**Nodal Stage N0**	**CCL5+**	78		n.s.	2.10	0.075	2.06	0.084
**Nodal Stage N1/N2**	**CCL5−**	14		n.s.		n.s.		n.s.
**Nodal Stage N1/N2**	**CCL5+**	38	0.45	**0.028**		n.s.		n.s.
**Nodal Stage NX**	**CCL5−**	6		n.s.		n.s.		n.s.
**Nodal Stage NX**	**CCL5+**	7		n.s.		n.s.		n.s.
**Basal**	**CCL5−**	16		n.s.		n.s.		n.s.
**Basal**	**CCL5+**	64		n.s.		n.s.		n.s.
**Luminal**	**CCL5−**	24		n.s.		n.s.		n.s.
**Luminal**	**CCL5+**	44		n.s.		n.s.	1.92	0.095
**Luminal EMT-p53-like**	**CCL5−**	6		n.s.		n.s.		n.s.
**Luminal EMT-p53-like**	**CCL5+**	11		n.s.		n.s.		n.s.
**-**				**Multivariate Cox Regression Analysis**				
		**N**	**OS**		**DSS**		**RFS**	
			**RR**	***p***	**RR**	***p***	**RR**	***p***
**All Patients**	**CCL5−**	51	0.30	**0.006**	0.36	**0.022**	0.41	**0.046**
**All Patients**	**CCL5+**	123		n.s.		n.s.		n.s.
**Tumor Stage 2**	**CCL5−**	10		n.d.		n.d.		n.d.
**Tumor Stage 2**	**CCL5+**	36		n.s.		n.s.		n.s.
**Tumor Stage 3 + 4**	**CCL5−**	41	0.28	**0.004**	0.32	**0.011**	0.36	**0.024**
**Tumor Stage 3 + 4**	**CCL5+**	87	0.65	0.098		n.s.		n.s.
**Nodal Stage N0**	**CCL5−**	31	0.22	**0.028**		n.s.		n.s.
**Nodal Stage N0**	**CCL5+**	78		n.s.	2.30	0.082	2.28	0.084
**Nodal Stage N1/N2**	**CCL5−**	14		n.s.		n.s.		n.s.
**Nodal Stage N1/N2**	**CCL5+**	38	0.44	**0.026**	0.50	0.091		n.s.
**Nodal Stage NX**	**CCL5−**	6		n.s.		n.s.		n.s.
**Nodal Stage NX**	**CCL5+**	7		n.s.		n.s.		n.s.
**Basal**	**CCL5−**	16		n.s.		n.s.		n.s.
**Basal**	**CCL5+**	64		n.s.		n.s.		n.s.
**Luminal**	**CCL5−**	24	0.25	**0.023**	0.29	**0.041**	0.23	**0.020**
**Luminal**	**CCL5+**	44		n.s.		n.s.		n.s.
**Luminal EMT-p53-like**	**CCL5−**	6		n.s.		n.s.		n.s.
**Luminal EMT-p53-like**	**CCL5+**	11		n.s.		n.s.		n.s.

Significant *p*-values are marked in bold face; n.s.: not significant; n.d.: not determined.

## Data Availability

All data are available in the manuscript and the Supplementary Materials. The detailed datasets used and analyzed during the present study are available from the corresponding author upon reasonable request.

## Supplementary Materials

The following supporting information can be downloaded: https://www.mdpi.com/article/10.3390/ijms25126325/s1.

## Abbreviations

BC	bladder cancer
CCL5	CC-chemokine-ligand 5
DSS	disease-specific survival
ICs	immune cells
IHC	immunohistochemistry
K–M analysis	Kaplan–Meier analysis
MIBC	Muscle-invasive bladder cancer
OS	overall survival
RFS	recurrence-free survival
TCs	tumor cells
