# Supplementary material for: Prognostic and Predictive Potential of CCL5 Expression in Muscle-Invasive Bladder Cancer Patients
Source: Int J Mol Sci. 2024 Jun 7;25(12):6325. doi: 10.3390/ijms25126325 (PMC11204343; doi:10.3390/ijms25126325)
Supplement: Supplementary file 1 [file ijms-25-06325-s001.zip › ijms-2982481-supplementary materials.pdf]

Suppl. Tables

Prognostic and Predictive Potential of CCL5 Expression for Muscle Invasive Bladder Cancer Patients

Cedric Smolka [1,2,†], Markus Eckstein [1,3,4,†], Rudolf Jung [1], Verena Lieb [2,3], Danijel Sikic [2,3,4], Robert Stöhr [1,3,4], Veronika Bahlinger [1], Simone Bertz [1,3], Astrid Kehlen [5], Arndt Hartmann [1,3,4], Bernd Wullich [2,3,4], Helge Taubert [2,3,4,*,‡] and Sven Wach [2,3,4,‡]

Table S1. CCL5 expression in TCs and in ICs of MIBC patients.

	N	CCL5 negative (in %)	CCL5 positive (in %)
Tumor cells (TCs)	175	100 (57.1)	75 (42.9)
Immune cells (ICs)	175	52 (29.7)	123 (70.3)

Table S2. Spearman's bivariate correlation between CCL5 staining in TCs or ICs and clinicopathological/molecular parameters in MIBC patients.

Parameter		CCL5 in TCs		CCL5 in ICs
**Gender**	r_s	0.011	r_s	0.035
	P	0.884	P	0.642
**Age at cystectomy**	r_s	0.067	r_s	0.008
	P	0.375	P	0.921
**pT**	r_s	0.019	r_s	-0.104
	P	0.806	P	0.170
**pN**	r_s	0.159	r_s	-0.036
	P	**0.036***	P	0.633
**OS**	r_s	-0.141	r_s	-0.068
	P	0.062	P	0.371
**DSS**	r_s	-0.134	r_s	0.152
	P	0.078	P	**0.045***
**Survival time**	r_s	-.195	r_s	0.234
	P	**0.010***	P	**0.002****
**time to recurrence**	r_s	-0.163	r_s	0.257
	P	**0.031***	P	**0.001****
**RFS**	r_s	-0.134	r_s	0.152
	P	0.078	P	**0.045***
**CCL5 in ICs/TCs**	r_s	0.184	r_s	0.184
	P	**0.015***	**P**	**0.015***
**Chemotherapy**	r_s	0.015	r_s	-0.006
	P	0.840	P	0.932
**molecular subtypes**	r_s	-0.070	r_s	-0.015
	P	0.359	P	0.844
**TILs**	r_s	-0.023	r_s	0.368
	P	0.763	P	**<0.001****
**CD3**	r_s	-0.012	r_s	0.301
	P	0.877	P	**<0.001****
**CD8**	r_s	-0.010	r_s	0.348
	P	0.893	P	**<0.001****
**CD56**	r_s	0.043	r_s	0.351
	P	0.569	P	**<0.001****
**CXCL9**	r_s	0.009	r_s	0.394
	P	0.911	P	**<0.001****

Characterization of TILs, CD3, CD8, CD56 and CXCL9 have been described previously [9]. Significant values are marked in bold. r_s=correlation coefficient, P=P-value (2-sided), *P<0.05, **P < 0.01.
